# Bergamot Polyphenol Fraction Exerts Effects on Bone Biology by Activating ERK 1/2 and Wnt/β-Catenin Pathway and Regulating Bone Biomarkers in Bone Cell Cultures

**DOI:** 10.3390/nu10091305

**Published:** 2018-09-14

**Authors:** Arturo Pujia, Cristina Russo, Samantha Maurotti, Roberta Pujia, Vincenzo Mollace, Stefano Romeo, Tiziana Montalcini

**Affiliations:** 1 Department of Medical and Surgical Science, University Magna Grecia, 88100 Catanzaro, Italy; pujia@unicz.it (A.P.); crusso@unicz.it (C.R.); samabiotec@yahoo.it (S.M.); romeo@unicz.it (S.R.); 2 Department of Health Science, University Magna Grecia, 88100 Catanzaro, Italy; robertapuj@gmail.com (R.P.); mollace@unicz.it (V.M.); 3 Department of Molecular and Clinical Medicine, University of Gothenburg, 40530 Gothenburg, Sweden; 4 Department of Clinical and Experimental Medicine, Nutrition Unit, University Magna Grecia, 88100 Catanzaro, Italy

**Keywords:** polyphenols, citrus fruits, Bergamot, osteoporosis, Saos-2, bone cells, β-catenin

## Abstract

Epidemiological studies show that fruit consumption may modulate bone mineral density. However, data regarding the effect of the *Citrus bergamia* Risso (Bergamot orange), a citrus fruit containing a high concentration of flavonoids, on bone health are still lacking. In this study, we investigated the effects of Bergamot polyphenols on the Wnt/β-catenin pathway in two distinct bone cell types (Saos-2 and MG63). Findings showed that exposure to 0.01 and 0.1 mg/mL doses upregulate β-catenin expression (*p* = 0.001), osteoblast differentiation markers (e.g., *RUNX2* and *COL1A*), and downregulate RANKL (*p* = 0.028), as compared to the control. Our results highlight, for the first time, that Bergamot polyphenols act on bone cells through the β-catenin pathway. In vivo studies are necessary to fully understand Bergamot’s role against bone resorption.

## 1. Introduction

Despite the wide range of pharmacological agents that reduce fracture risks [1,2,3,4,5,6,7,8], safety concerns have arisen regarding long-term osteoporosis pharmacotherapy. The long-term use of bisphosphonates is associated with an increased risk of atypical fractures [9], upper gastrointestinal adverse events [10], and osteonecrosis of the jaw (ONJ) [11]. A recent report has demonstrated that denosumab can also induce ONJ [12]. The use of hormone therapy (HT) for the prevention of osteoporosis in elderly postmenopausal women is currently under review, due to the increased risk of breast cancer and thromboembolic disease [13,14]. Because of the occurrence of osteosarcoma in toxicity studies, the use of teriparatide is limited to 24 months of treatment [15]. Finally, strontium ranelate has been associated with rare cases of hypersensitivity drug reaction with eosinophilia and systemic symptoms (DRESS) [16]. There is general consensus that the benefits of treatment far outweigh any risks associated with long-term treatment [17], but it is evident that there is an urgent need to find new and cost-efficient ways to prevent the development of osteoporosis. 

Fruits and vegetables are rich in a variety of bioactive compounds, with antioxidant properties, that may effectively improve bone health [18]. Citrus fruits are important due to their high polyphenol content. However, to date, the effects of polyphenols, as nutraceuticals, on bone cell metabolism have scarcely been studied. Additionally, little research has focused on the potential effects of the polyphenols from *Citrus bergamia* Risso (Bergamot orange) on bone. Accordingly, we investigated the role of the Bergamot polyphenol fraction (BPF) in vitro on the intracellular pathways, cell proliferation, differentiation, and expression of the bone extracellular matrix proteins, in two distinct bone cell models (Saos-2 and MG63). Because the Wnt/β-catenin pathway is a key target for developing drugs against bone loss [19], we mainly focused on verifying whether BPF acts at this level.

## 2. Materials and Methods

### 2.1. Cell Culture

The human osteoblast-like cell lines, Saos-2 and MG63, were obtained from American Type Culture Collection ATCC (Italy Office, via Venezia 23, 20,099 Sesto San Giovanni, Milan, Italy) and maintained in McCoy’s 5A and DMEM (Gibco, Carlsbad, CA, USA), respectively. They were supplemented with 15% and 10% fetal bovine serum (Gibco, Carlsbad, CA, USA), respectively, with 1% penicillin streptomycin (PAA, Linz, Austria), at 37 °C in 5% CO_2_, harvested by trypsinization, and subcultured twice weekly. To obtain more differentiated cell lines, Saos-2 and MG63 cells were incubated with dexamethasone 10 nM (Sigma, St. Louis, MO, USA) in all of the experiments.

### 2.2. Western Blotting and β-Catenin Knockdown

Saos-2 cells were seeded at a density of 200,000 cells/well in 6-well dishes, and 500,000 cells/well in 100 mm culture dishes. MG63 cells were seeded at a density of 100,000 cells/well in 6-well dishes. Cells were grown in serum-free medium and incubated with BPF 0.001; 0.001; 0.1 mg/mL for 10 min or 24 h.

BPF, as previously prepared and characterized for polyphenol content [20], was provided by Herbal and Antioxidant Derivatives srl. (Polistena, RC, Italy). The main flavonoids identified in BPF were neoeriocitrin (370 ppm), naringin (520 ppm), and neohesperidin (310 ppm).

Moreover, Saos-2 cells were transfected with β-catenin small interfering RNA (siRNA) or with negative control (scramble) siRNA (Ambion-Life Technologies by Thermo Fisher Scientific, Rockford, IL, USA), with Lipofectamine 3000 Reagent, according to the manufacturer’s instructions. The optimum concentration for silencing β-catenin expression was 10 nM. Maximum reduction of catenin expression occurred at 48 h post-transfection.

Cells were lysed in Mammalian Protein Extraction Reagent (M-PER) (Pierce, Thermo Fisher Scientific). Western blot analysis was performed according to standard procedures. The following antibodies were used: rabbit anti β-catenin (19,807), rabbit anti-Runt-related transcription factor 2 (RUNX2) (12,556), rabbit anti-p Extracellular Signal-regulated Kinase (ERK)1/2 (9101) and mouse anti-β-actin (3700), by Cell Signaling Technology (Beverly, MA, USA); rabbit anti-type I collagen (COL1) (HPA011795) and rabbit anticalnexin (C4731), from Sigma Aldrich (St. Louis, MO, USA); mouse anti-receptor activator of nuclear factor-κB ligand (RANKL) (sc-377079), by Santa Cruz Biotechnology (USA); rabbit anti-OPG, by Abcam (Cambridge, UK); and mouse anti-Alkaline phosphatase (ALP), by Abcam (Cambridge).

### 2.3. Real Time-PCR

Saos-2 cells were seeded at a density of 200,000 cells/well in 6-well dishes, and 500,000 cells/well in 100 mm culture dishes. Cells were grown in serum-free medium and incubated with BPF 0.001; 0.001; 0.1 mg/mL for 24 h. Total RNA of cells were extracted with Trizol reagent (Life technologies, UK) according to manufacturer’s instructions. cDNA was synthesized from 1 µg total RNA, using High-Capacity cDNA Reverse Transcription Kit (Applied Biosystems, Foster City, CA, USA). mRNA expression of RANKL, osteoprotegerin, RUNX2, type I collagen, and β-actin were quantified by real time-PCR using SYBR^®^ Green dye (SYBR^®^ Green PCR Master Mix, Applied Biosystems, Foster City, CA, USA) (see Table 1).

### 2.4. ALP Activity

Saos-2 cells were seeded at a density of 50,000 cells/well in 24-well dishes. Cells were cultured in osteogenic medium, supplemented with b-glycerophosphate 7.5 mM (SERVA, Heidelberg, Germany), ascorbic acid 50 mg/mL (Santa Cruz, CA, USA) and dexamethasone 10 nM, and incubated with BPF 0.001; 0.001; 0.1 mg/mL for 24 h. Cells were lysed with ice cold 50 mM Tris-HCl solution with 0.05% Triton X-100. Cells were then centrifuged at 1000 *g*, 4 °C for 15 min. Protein concentration was determined using Bradford assay, and ALP activity was determined by p-nitrophenyl phosphate (pNPP) colorimetric method (WAKO Chemicals USA, Richmond, VA, USA).

### 2.5. Cell Viability Assay

To evaluate cell viability, Saos-2 cells were seeded at a density of 10,000 cells/well in 96-well plates. Cells were grown in serum-free medium and incubated with BPF 0.001; 0.001; 0.1 mg/mL for 24 h. Cell viability was determined by 3-(4,5-dimethylthiazol-2-yl)-2,5-diphenyltetrazolium bromide (MTT) assay. Briefly, MTT (Sigma, St. Louis, MO, USA) solution (5 mg/mL) was added to each well, and incubated at 37 °C for 4 h. The supernatants were then removed and replaced by 100 mL DMSO. The optical density (OD) was measured at a wavelength of 570 nm.

### 2.6. Cell Proliferation

Saos-2 and MG63 cells were seeded at a density 200,000 and 100,000 cells/well, respectively, in 6-well dishes. Cells were grown in serum-free medium, and incubated with BPF 0.001; 0.001; 0.1 mg/mL for 24 h. Then, cell number was determined using the Nucleo counter NC-100 (Chemometec A/S, Lillerød, Denmark).

### 2.7. Statistical Analysis

Data are represented as mean ± standard deviation of at least three independent experiments, and analyzed using two-tailed Student’s *t*-test and linear regression. *p*-values less than 0.05 were considered significant. Statistical analysis was performed with GraphPad Prism 5.0.

## 3. Results

### 3.1. BPF Does Not Act on Osteoblast Viability and Proliferation In Vitro

**Figure 1:**
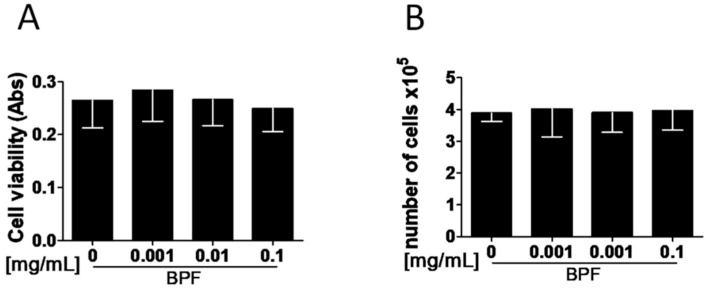
BPF does not increase viability and cell proliferation of Saos-2 cells. Semi-confluent cultures of human osteoblast-like cells (Saos-2) incubated with BPF 0.001-0.01-0.1 mg/mL for 24 h. (**A**) Cell viability determined by MTT assay. (**B**) Cell proliferation determined by counting the number of cells in each well. Data are represented as mean ± SD. Abbreviations: BPF, Bergamot Polyphenol fraction; MTT assay, 3-(4,5-dimethylthiazol-2-yl)-2,5-diphenyltetrazolium bromide assay.

To test the hypothesis that BPF increases osteoblast proliferation, Saos-2 cells were incubated with different doses of BPF for 24 h and cell viability was measured by 3-(4,5-dimethylthiazol-2-yl)-2,5-diphenyltetrazolium bromide (MTT) assay. There were no differences in Saos-2 cell viability between all concentrations of BPF (Figure 1A). Then, cell proliferation was determined by counting the cell number. There were no differences between all doses of BPF (Figure 1B).

### 3.2. BPF Induces Higher pERK1/2 Levels in Saos-2

**Figure 2:**
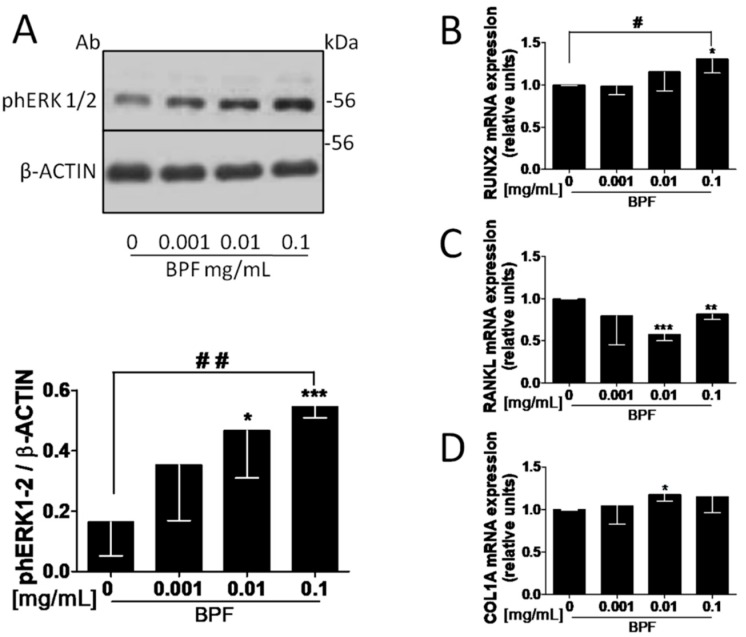
BPF increases phERK 1/2 protein levels and regulates RUNX2, RANKL and COL1A mRNA expression levels on Saos-2 cells. (**A**) Semi-confluent cultures of human osteoblast-like cells (Saos-2) incubated with BPF 0.001-0.01-0.1 mg/mL for 10 min. Cell proteins were analyzed by Western blotting with antibodies specific to phosphorylated ERK1/2 and β-actin. Semi-confluent cultures of human osteoblast-like cells (Saos-2) were incubated with BPF 0.001-0.01-0.1 mg/mL for 24 h. Then, mRNA expression levels of (**B**) RUNX2, (**C**) RANKL and (**D**) COL1A were measured by RT-Pcr. Data were analyzed using the 2^−ΔΔCq^ method and normalized to β-actin. Data are represented as mean ± SD. Statistical analysis: Student’s *t*-test vs. 0 * *p* < 0.05; ** *p* < 0.01; *** *p* < 0.001; Linear regression ^#^ *p* < 0.05; ^##^ *p* < 0.01. Abbreviations: BPF, Bergamot Polyphenol Fraction; RUNX2, Runt-related transcription factor 2; RANKL, Receptor Activator of Nuclear factor-κB Ligand; COL1A, Type 1A collagen.

To test the hypothesis that BPF activates ERK1/2, Saos-2 cells were grown in medium containing 10 nM dexamethasone, and then incubated with BPF at concentrations of 0.001, 0.01 and 0.1 mg/mL (Figure 1). BPF incubation increased ERK1/2 phosphorylation, in a dose dependent manner, compared to the control untreated cells (*p* = 0.001; Figure 2A). 

### 3.3. BPF Increases β-Catenin, RUNX2 and Intracellular COL1A Proteins and Decreases Both the RANKL and Extracellular COL1A Protein Levels in Saos-2

Osteoblastic Saos2 cells were exposed to 0.001, 0.01 and 0.1 mg/mL BPF. Only the 0.1 mg/mL dose increased RUNX2 mRNA levels, compared to the control (*p* = 0.031; Figure 2B).

RANKL mRNA levels decreased and COL1A mRNA levels increased at 0.01 mg/mL, compared to the control (*p* = 0.0007 and *p* = 0.016, respectively; Figure 2C,D). In addition, RANKL mRNA levels decreased and COL1A mRNA did not change at 0.1 mg/mL, compared to the control (*p* = 0.006) (Figure 2C,D).

**Figure 3:**
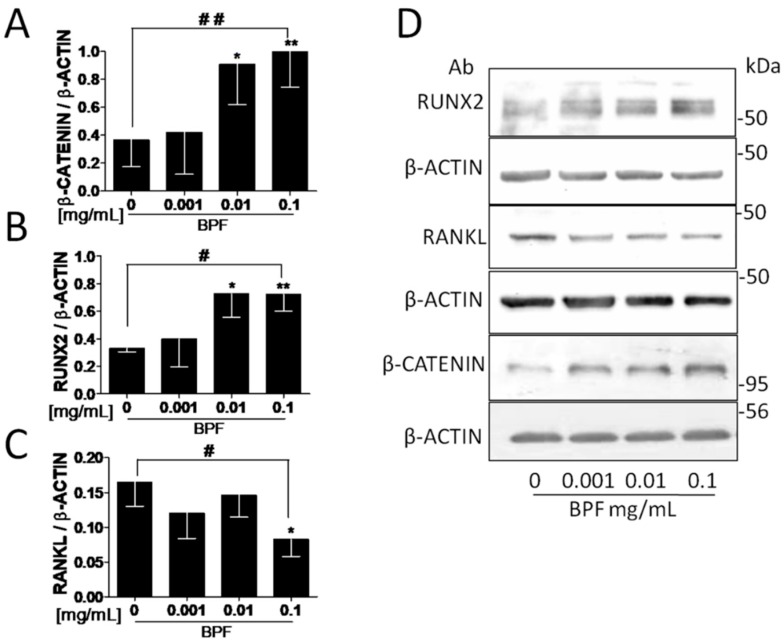
BPF increases β-catenin and RUNX2 and decreases RANKL protein levels on Saos-2 cells. Semi-confluent cultures of human osteoblast-like cells (Saos-2) were incubated with BPF 0.001-0.01-0.1 mg/mL for 24 h. Cell proteins were analyzed by Western blotting (**D**) with antibodies specific to (**A**) β-catenin, (**B**) RUNX2, (**C**) RANKL and β-actin. Data are represented as mean ± SD. Statistical analysis: Student’s *t*-test vs. 0 * *p* < 0.05; ** *p* < 0.01. Linear regression ^#^ *p* < 0.05; ^##^ *p* < 0.01. Abbreviations: BPF, Bergamot Polyphenol Fraction; RUNX2, Runt-related transcription factor 2; RANKL, Receptor Activator of Nuclear factor-κB Ligand.

Exposure to increasing doses of BPF resulted in higher protein levels of β-catenin and RUNX2 (*p* = 0.001 and *p* = 0.039, respectively), and lower levels of RANKL (*p* = 0.028), than the control (Figure 3A–C, respectively).

**Figure 4:**
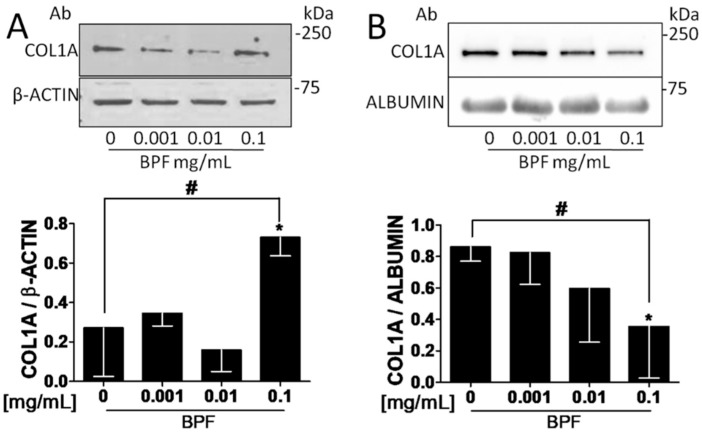
BPF increases intracellular COL1A protein levels and decreases extracellular COL1A protein levels on Saos-2 cells. Semi-confluent cultures of human osteoblast-like cells (Saos-2) were incubated with BPF 0.001-0.01-0.1 mg/mL for 24 h. Cell and medium proteins ((**A**,**B**) respectively) were analyzed by Western blotting, with antibodies specific to COL1A, β-actin and Albumin. Data are represented as mean ± SD. Statistical analysis: Student’s *t*-test vs. 0 * *p* < 0.05. Linear regression ^#^ *p* < 0.05. Abbreviations: BPF, Bergamot Polyphenol fraction; COL1A, Type 1A collagen.

Figure 4A shows that intracellular COL1A production increased at the 0.1 mg/mL dose, compared to the control (*t*-test, *p* = 0.038). Whereas Figure 4B shows that the extracellular COL1A secretion was markedly suppressed in a dose dependent manner (up to 45%; *p* = 0.011).

Osteoprotegerin mRNA, ALP activity, and their proteins, did not change after stimulation, with all concentrations of BPF (Supplementary Figures S1A,B and S2A,B).

### 3.4. RNA Interference Shows that RUNX2 and RANKL Expression Is Mediated by β-Catenin

We next attempted to confirm the role of BPF on β-catenin, by silencing its expression using siRNA, and assessed its effects on RUNX2 and RANKL expression in vitro.

**Figure 5:**
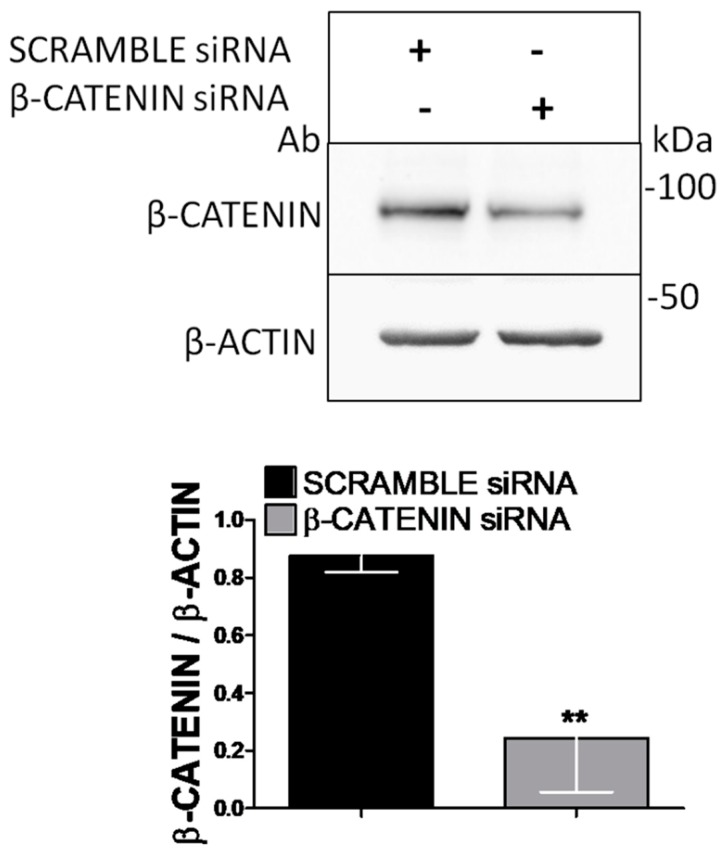
The effect of β-catenin knockdown on Saos-2 cells. Semi-confluent cultures of human osteoblast-like cells (Saos-2) were incubated with 10 nM β-catenin or scrambled siRNA for 48 h. Cell proteins were analyzed by Western blotting, with antibodies specific to β-catenin and β-actin. Data are represented as mean ± SD. Statistical analysis: Student’s *t*-test vs. scrambled siRNA ** *p* < 0.01. Abbreviations: BPF, Bergamot Polyphenol Fraction.

siRNA effectively suppressed β-catenin protein level (*p* = 0.005), whereas the scrambled siRNA did not influence the protein level (Figure 5).

**Figure 6:**
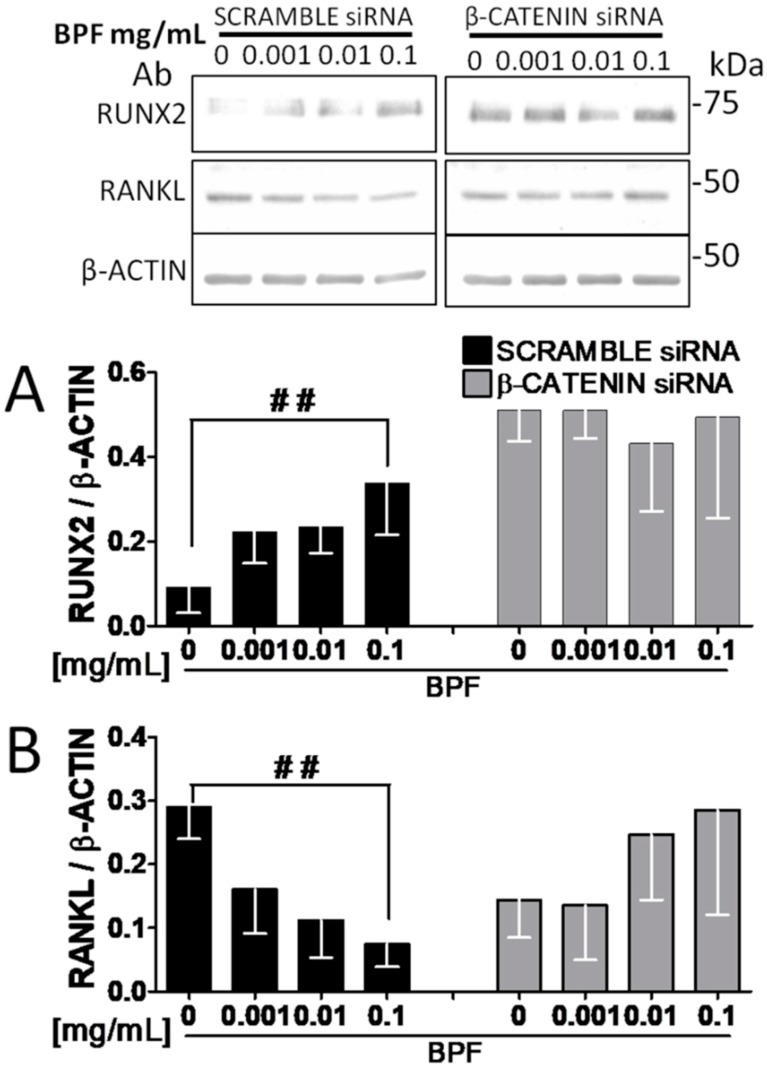
Reverted effects on both the RUNX2 and RANK-L protein expression with BPFon β-catenin knockdown Saos-2 cells. Semi-confluent cultures of human osteoblast-like cells (Saos-2) were pretreated with scrambled or β-catenin siRNA for 48 h, then incubated with BPF 0.001-0.01-0.1 mg/mL for 24 h. Cell proteins were analyzed by Western blotting, with antibodies specific to (**A**) RUNX2, (**B**) RANKL and β-actin. Data are represented as mean ± SD. Statistical analysis: Linear regression; ^##^ *p* < 0.01. Abbreviations: BPF, Bergamot Polyphenol Fraction; RUNX2, Runt-related transcription factor 2; RANKL, Receptor Activator of Nuclear factor-κB Ligand.

As expected, inhibition of β-catenin by siRNA, in the presence of BPF, reverted the effects on both the RUNX2 and RANKL protein expression, compared to the control cells transfected with a scrambled siRNA, where RUNX2 increased and RANKL decreased (Figure 6A,B).

### 3.5. BPF Increases β-Catenin Protein Levels in MG63.

**Figure 7:**
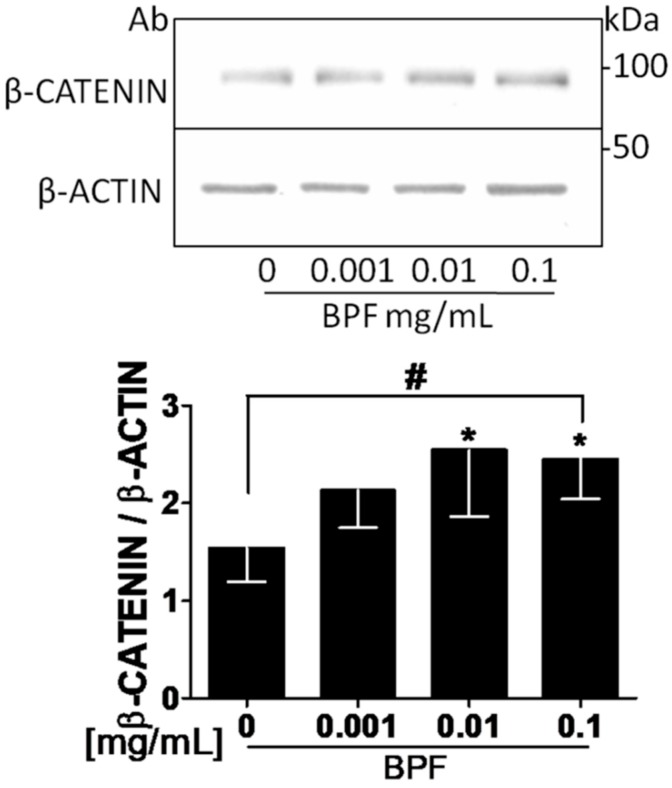
BPF increases β-catenin protein levels in MG-63 cells. Semi-confluent cultures of human osteoblast-like cells (MG-63) were incubated with BPF 0.001-0.01-0.1 mg/mL for 24 h. Cell proteins were analyzed by Western blotting with antibodies specific to β-catenin and β-actin. Data are represented as mean ± SD. Statistical analysis: Student’s *t*-test vs. 0 * *p* < 0.05. Linear regression ^#^ *p* < 0.05. *Abbreviations*: BPF, Bergamot Polyphenol Fraction.

Since the MG63 cell line provides a useful model for studying events associated with the early osteoblastic differentiation stage [21], MG63 cells were incubated with different doses of BPF for 24 h. Then cell proliferation was determined by counting the cell number. There were no differences in the cell proliferation at all doses of BPF (Supplementary Figure S3), whereas the β-catenin protein expression significantly increased, compared to the control untreated cells (*p* = 0.012; Figure 7).

## 4. Discussion

This study, which is the first to directly investigate the effects of Bergamot polyphenols in bone cell models, shows that β-catenin, ERK 1/2, RUNX2, and intracellular COL1A proteins are upregulated by exposure to increasing doses of BPF. In addition, the expression of RANKL, which is a known stimuli for increasing osteoclast activity, is reduced in these cells, suggesting that BPF down-regulates RANKL, and may have a potential role in bone resorption.

To identify potential differences in β-catenin expression during cell maturation, we performed our experiments in two distinct bone cell types. Saos-2 cells represent a valuable model for studying events associated with the late osteoblastic differentiation stage in human cells [21], whereas MG63 cells are associated with the early stage in human cells. We found that BPF induced β-catenin expression in both MG63 and Saos-2 cells. This is one of the key findings of this study. 

It is known that a high fruit and vegetable intake is associated with a lower risk of osteoporosis [18,22,23,24]. However, to date, the effects of citrus fruit polyphenols on bone cell metabolism have scarcely been studied. In one study, hesperidin, which is a citrus flavonoid, helped to inhibit bone loss of ovariectomized mice [25]. In other studies, because of their flavonoid content, orange and grapefruit juices positively affected serum antioxidant capacity, bone strength, and the fracture risk of ovariectomized rats [26,27]. Bergamot (*Citrus bergamia* Risso) differs from other citrus fruits because of its particularly high flavonoid concentration [28]. Bergamot is an endemic plant of Calabria, in southern Italy, with a unique profile of flavonoid and flavonoid glycosides in its juice, such as neoeriocitrin, neohesperidin, naringin, rutin, neodesmin, rhoifolin and poncirin. It has been demonstrated that BPF, with its antioxidant properties, has pleiotropic beneficial effects in preventing and reducing heart muscle damage [29]. Since oxidative stress is involved in the pathogenesis of osteoporosis, we investigated the effects of BPF on bone cell metabolic markers in vitro, by two distinct osteoblast models.

In bone, the extracellular matrix (ECM) plays an important role in osteoblast function. It is known that ECM–integrin interaction leads to the activation of the MAPKs, ERK1 and ERK2, resulting in increased RUNX2 phosphorylation, and consequently the expression of osteoblast differentiation genes, such as collagen, osteocalcin, osteopontin and bone sialoprotein [30,31]. The β-catenin signaling system functions as a transcriptional activator, and plays a crucial role in stabilizing intercellular adhaerens junctions. It has been demonstrated, in one animal model, that interference with cadherin–β-catenin interactions leads to a reduced bone mass [32]. ERK is an important factor in osteoblast differentiation, and RUNX2 is a bone specific transcription factor that is tightly regulated during the late mineralization stage of osteoblast differentiation [33]. ERK inhibition results in the suppression of differentiation markers [34]. Syringetin, the main flavonoid present in red grapes, may be beneficial in stimulating osteoblastic activity, by involving the ERK1/2 signaling pathway [35]. In another study, naringin treatment enhanced both transcriptional and translational levels of the β-catenin signaling of UMR-106 cells, and improved bone development in OVX mice, by stabilizing β-catenin through AMPK and Akt signaling [36].

In line with these previous investigations [30,31,32,33,34,35,36], we found that BPF increased β-catenin, ERK1/2, and RUNX2 protein expression in a dose-dependent manner (Figure 3 and Figure 4).

We used siRNAs to suppress bone matrix protein expression. As shown in previous studies [37,38,39], RNA interference, using siRNA, is a useful biological strategy to study bone loss pathogenesis, and is a novel approach to treat several diseases. As expected, the inhibition of β-catenin by siRNA, in the presence of BPF, reversed the effects on RUNX2 and RANKL protein expression.

Taken together, our experiments with RNA interference confirm the central role of Wnt/β-catenin in osteoblast gene expression, and that BPF acts through this pathway.

COL1A, the main component of a bone matrix, plays a key role in bone, in transferring stress and resisting against deformation and fractures [40]. However, in our study we found a significant reduction in the secretion of the extracellular COL1A (Figure 4B). This finding may confirm that a negative feedback mechanism exists to preserve bone tissue homeostasis. It has been demonstrated that serum TGF-β1, which is produced by osteoblast, is reduced in osteoporotic men [41], and that TGF-β1 is downregulated by extracellular COL1 and COL2 [42]. Thus, reduced extracellular COL1, by upregulating TGF-β1, might protect against osteoarticular diseases. Our experiments also show that BPF produced higher amounts of intracellular COL1A than the control cells (Figure 4A). We hypothesize that BPF might promote bone repair through balancing collagen synthesis. However, since posttranslational processes are strongly involved in the formation of COL1 fibers, we believe that estrogen, vitamin D, and age-related effects on collagen secretion should be investigated in detail in future studies.

In this study, the incubation time and doses used were chosen based on previous reports [29,43,44]. In addition, since some natural polyphenols produce H_2_O_2_, during the so-called autoxidation process of polyphenols, and H_2_O_2_ acts as a second messenger modulating gene expression [45], we chose the short incubation time of 10 min.

Flavanone distribution within different citrus species can be quite distinctive. The active ingredients in Bergamot are mainly naringin, neohesperidin and neoeriocitrin. *C. bergamia* contains 2.23, 1.60 and 1.38 mg/100 mL of naringin, neohesperidin and neoeriocitrin, respectively, whereas *C. aurantium* (bitter orange) contains 1.97, 0.87 and 0.77 mg/100 mL of these same flavanones, respectively, and *C. aurantifolia* (lime) only contains 0.01 mg/100 mL of neoeriocitrin [46]. It is evident that Bergamot is characterized by its unique profile of flavanones, and the large amounts of them. Furthermore, Bergamot contains the rare brutieridin and melitidin flavonoids.

Most of the beneficial effects found for citrus flavonoids were based on animal and in vitro studies, which were crucial in explaining mechanisms of these components. Unfortunately, very limited clinical studies have been conducted on citrus flavonoids, or citrus fruit juices, in relation to possible benefits on bone. Furthermore, the amount of polyphenols, flavonoids or other bioactive components cannot be determined from these studies. There is also a significant lack of information regarding clinical studies with pure naringin, neohesperidin and neoeriocitrin. In one study, BFP was safely used at a dose of 500 and 1000 mg/day [20]. In another study, Fang et al. [47] reported plasma concentrations, in rats, of 3.8, 0.23 and 43.5 μg/mL for naringin, naringenin and naringenin glucuronide, respectively, after an oral administration of 746·7 mg/kg naringin as a pure compound. Because we tested BPF from 1 to 100 μg/mL, and in line with these previous studies, we may predict the in vivo exposure conditions. However, we recognize that, when extrapolating in vitro models to in vivo scenarios, additional evidence is needed.

Our results may have other clinical implications. Loss of Wnt/beta-catenin pathway activity may contribute to osteosarcoma development [48]. Osteosarcoma is the most common malignant bone cancer. Thus, carefully designed studies are needed to investigate the anticancer functions of BPF.

## 5. Conclusions

Our results highlight, for the first time, a potential positive effect of polyphenols from Bergamot, a citrus fruit containing a high flavonoid concentration, on osteoblast differentiation and production of collagen. BPF also reduces RANKL expression, suggesting that it may inhibit osteoclastogenesis. Further studies are necessary to confirm our findings.

## Figures and Tables

**Table 1:** Real time-PCR primer sequences.

Gene	Forward	Reverse
RUNX2	5′-TTACTTACACCCCGCCAGTC-3′	5′-TATGGAGTGCTGCTGGTCTG-3′
RANKL	5′-AGAGCGCAGATGGATCCTAA-3′	5′-TTCCTTTTGCACAGCTCCTT-3′
COL1A	5′-CCCCAGCCCACAAAGAGTCTA-3′	5′-CTGTACGCAGGTGATTGGTG-3′
Osteoprotegerin	5′-TGCAGTACGTCAAGCAGGAG-3′	5′-GTGTCTTGGTCGCCATTTTT-3′
β-actin	5′-GACTGTGACGAGTTGGCTGA-3′	5′-CTGGAGAGGAGCAGAACTGG-3′

## Supplementary Materials

The following are available online at http://www.mdpi.com/2072-6643/10/9/1305/s1. This work includes supplementary data (Supplementary Figures S1–S3). (Supplementary Figure S1) BPF does not increase Osteoprotegerin mRNA and protein levels on Saos-2 cells. Semi-confluent cultures of human osteoblast-like cells (Saos-2) were incubated with BPF 0.001-0.01-0.1 mg/mL for 24 h. (A) mRNA expression levels of Osteoprotegerin were measured by RT-Pcr. Data were analyzed using the 2^−ΔΔCq^ method and normalized to β-actin. (B) Cell proteins were analyzed by Western blotting antibodies specific to Osteoprotegerin and β-actin. Data are represented as mean ± SD. Abbreviations: BPF, Bergamot Polyphenol Fraction. (Supplementary Figure S2) BPF does not increase ALP protein levels and activity on Saos-2 cells. Semi-confluent cultures of human osteoblast-like cells (Saos-2) were incubated with BPF 0.001-0.01-0.1 mg/mL for 24 h. (A) Cell proteins were analyzed by Western blotting antibodies specific to ALP and β-actin. (B) ALP activity was measured by pNPP method. Data are represented as mean ± SD. Abbreviations: BPF, Bergamot Polyphenol Fraction; ALP, Alkaline Phosphatase; pNPP, Para-Nitrophenyl Phosphate. (Supplementary Figure S3) BPF does not increase cell proliferation in MG-63 cells. Semi-confluent cultures of human osteoblast-like cells (MG-63) were incubated with BPF 0.001-0.01-0.1 mg/mL for 24 h. Cell proliferation was determined by counting the number of cells in each well. Data are represented as mean ± SD. Abbreviations: BPF, Bergamot Polyphenol Fraction.

## Abbreviations

ALP	Alkaline phosphatase
BPF	Bergamot polyphenol fraction
ERK	Extracellular Signal-regulated Kinase
MTT	3-(4,5-dimethylthiazol-2-yl)-2,5-diphenyltetrazolium bromide
ONJ	osteonecrosis of the jaw
RANKL	receptor activator of nuclear factor-κB ligand
RUNX2	Runt-related transcription factor 2
siRNA	small interfering RNA
COL1	type I collagen

## References

[1] Cummings S.R., Black D.M., Thompson D.E., Applegate W.B., Barrett-Connor E., Musliner T.A., Palermo L., Prineas R., Rubin S.M., Scott J.C. (1998). Effect of alendronate on risk of fracture in women with low bone density but without vertebral fractures: Results from the Fracture Intervention Trial. JAMA.

[2] McClung M.R., Geusens P., Miller P.D., Zippel H., Bensen W.G., Roux C., Adami S., Fogelman I., Diamond T., Eastell R. (2001). Effect of risedronate on the risk of hip fracture in elderly women. Hip Intervention Program Study Group. N. Engl. J. Med..

[3] Harris S.T., Watts N.B., Genant H.K., McKeever C.D., Hangartner T., Keller M., Chesnut C.H., Brown J., Eriksen E.F., Hoseyni M.S. (1999). Effects of risedronate treatment on vertebral and non vertebral fractures in women with postmenopausal osteoporosis: A randomized controlled trial. Vertebral Efficacy with Risedronate Therapy (VERT) Study Group. JAMA.

[4] Cauley J.A., Seeley D.G., Ensrud K., Ettinger B., Black D., Cummings S.R. (1995). Estrogen replacement therapy and fractures in older women. Study of Osteoporotic Fractures Research Group. Ann. Intern. Med..

[5] Black D.M., Delmas P.D., Eastell R., Reid I.R., Boonen S., Cauley J.A., Cosman F., Lakatos P., Leung P.C., Man Z. (2007). Once-year zoledronic acid for treatment of postmenopausal osteoporosis. N. Engl. J. Med..

[6] Neer R.M., Arnaud C.D., Zanchetta J.R., Prince R., Gaich G.A., Reginster J.Y., Hodsman A.B., Eriksen E.F., Ish-Shalom S., Genant H.K. (2001). Effect of parathyroid hormone (1–34) on fractures bone mineral density in postmenopausal women with osteoporosis. N. Engl. J. Med..

[7] Cummings S.R., San Martin J., McClung M.R., Siris E.S., Eastell R., Reid I.R., Delmas P., Zoog H.B., Austin M., Wang A. (2009). Denosumab for prevention of fractures in postmenopausal women with osteoporosis. N. Engl. J. Med..

[8] Delmas P.D., Ensrud K.E., Adachi J.D., Harper K.D., Sarkar S., Gennari C., Reginster J.Y., Pols H.A., Recker R.R., Harris S.T. (2002). Efficacy of raloxifene on vertebral fracture risk reduction in postmenopausal women with osteoporosis: Four-year results from a randomized clinical trial. J. Clin. Endocrinol. Metab..

[9] Schilcher J., Michaëlsson K., Aspenberg P. (2011). Bisphosphonate use and a typical fractures of the femoral shaft. N. Engl. J. Med..

[10] Lowe C.E., Depew W.T., Vanner S.J., Paterson W.G., Meddings J.B. (2000). Upper gastrointestinal toxicity of alendronate. Am. J. Gastroenterol..

[11] Zhang X., Hamadeh I.S., Song S., Katz J., Moreb J.S., Langaee T.Y., Lesko L.J., Gong Y. (2016). Osteonecrosis of the Jaw in the United States Food and Drug Administration’s Adverse Event Reporting System (FAERS). J. Bone Miner. Res..

[12] Khan A.A., Morrison A., Hanley D.A., Felsenberg D., McCauley L.K., O’Ryan F., Reid I.R., Ruggiero S.L., Taguchi A., Tetradis S. (2015). Diagnosis management of osteonecrosis of the jaw: A systematic review international consensus. J. Bone Miner. Res..

[13] Rossouw J.E., Anderson G.L., Prentice R.L., LaCroix A.Z., Kooperberg C., Stefanick M.L., Jackson R.D., Beresford S.A., Howard B.V., Johnson K.C. (2002). Risks benefits of estrogen plus progestin in healthy postmenopausal women: Principal results From the Women’s Health Initiative randomized controlled trial. JAMA.

[14] Hulley S., Grady D., Bush T., Furberg C., Herrington D., Riggs B., Vittinghoff E. (1998). Randomized trial of estrogen plus progestin for secondary prevention of coronary heart disease in postmenopausal women. Heart and Estrogen/progestin Replacement Study (HERS) Research Group. JAMA.

[15] Jolette J., Wilker C.E., Smith S.Y., Doyle N., Hardisty J.F., Metcalfe A.J., Marriot T.B., Fox J., Wells D.S. (2006). Defining a non carcinogenic dose of recombinant human parathyroid hormone 1–84 in a 2-year study in Fischer 344 rats. Toxicol. Pathol..

[16] Musette P., Brandi M.L., Cacoub P., Kaufman J.M., Rizzoli R., Reginster J.Y. (2010). Treatment of osteoporosis: Recognizing and managing cutaneous adverse reactions and drug-induced hypersensitivity. Osteoporos. Int..

[17] Schousboe J.T. (2013). Adherence with medications used to treat osteoporosis: Behavioral insights. Curr. Osteoporos. Rep..

[18] Shen C.L., von Bergen V., Chyu M.C., Jenkins M.R., Mo H., Chen C.H., Kwun I.S. (2012). Fruits and dietary phytochemicals in bone protection. Nutr. Res..

[19] Lerner U.H., Ohlssonm C. (2015). The WNT system: Background its role in bone. J. Intern. Med..

[20] Gliozzi M., Walker R., Muscoli S., Vitale C., Gratteri S., Carresi C., Musolino V., Russo V., Janda E., Ragusa S. (2013). Bergamot polyphenolic fraction enhances rosuvastatin-induced effect on LDL-cholesterol, LOX-1 expression and protein kinase B phosphorylation in patients with hyperlipidemia. Int. J. Cardiol..

[21] McQuillan D.J., Richardson M.D., Bateman J.F. (1995). Matrix deposition by a calcifying human osteogenic sarcoma cell line (SAOS-2). Bone.

[22] Qiu R., Cao W.T., Tian H.Y., He J., Chen G.D., Chen Y.M. (2017). Greater Intake of Fruit and Vegetables Is Associated with Greater Bone Mineral Density and Lower Osteoporosis Risk in Middle-Aged and Elderly Adults. PLoS ONE.

[23] Byberg L., Bellavia A., Orsini N., Wolk A., Michaëlsson K. (2015). Fruit and vegetable intake and risk of hip fracture: A cohort study of Swedish men and women. J. Bone Miner. Res..

[24] Zalloua P.A., Hsu Y.H., Terwedow H., Zang T., Wu D., Tang G., Li Z., Hong X., Azar S.T., Wang B. (2007). Impact of seafood and fruit consumption on bone mineral density. Maturitas.

[25] Chiba H., Uehara M., Wu J., Wang X., Masuyama R., Suzuki K., Kanazawa K., Ishimi Y. (2003). Hesperidin, a citrus flavonoid, inhibits bone loss and decreases serum and hepatic lipids in ovariectomized mice. J. Nutr..

[26] Deyhim F., Garica K., Lopez E., Gonzalez J., Ino S., Garcia M., Patil B.S. (2006). Citrus juice modulates bone strength in male senescent rat model of osteoporosis. Nutrition.

[27] Horcajada-Molteni M.N., Crespy V., Coxam V., Davicco M.J., Rémésy C., Barlet J.P. (2000). Rutin inhibits ovariectomy-induced osteopenia in rats. J. Bone Miner. Res..

[28] Mollace V., Sacco I., Janda E., Malara C., Ventrice D., Colica C., Visalli V., Muscoli S., Ragusa S., Muscoli C. (2011). Hypolipemic and hypoglycaemic activity of bergamot polyphenols: From animal models to human studies. Fitoterapia.

[29] Carresi C., Musolino V., Gliozzi M., Maiuolo J., Mollace R., Nucera S., Maretta A., Sergi D., Muscoli S., Gratteri S. (2018). Anti-oxidanteffect of bergamot polyphenolic fraction counteract doxorubicin-induced cardiomyopathy: Role of autophagy and c-kit(pos) CD45(neg) CD31(neg) cardiac stem cell activation. J. Mol. Cell. Cardiol..

[30] Ge C., Xiao G., Jiang D., Franceschi R.T. (2007). Critical role of the extracellular signal-regulated kinase-MAPK pathway in osteoblast differentiation and skeletal development. J. Cell Biol..

[31] Hamidouche Z., Fromigué O., Ringe J., Häupl T., Vaudin P., Pagès J.C., Srouji S., Livne E., Marie P.J. (2009). Priming integrin alpha5 promotes human mesenchymal stromal cell osteoblast differentiation and osteogenesis. Proc. Natl. Acad. Sci. USA.

[32] Castro C.H., Shin C.S., Stains J.P., Cheng S.L., Sheikh S., Mbalaviele G., Szejnfeld V.L., Civitelli R. (2004). Targeted expression of a dominant-negative N-cadherin in vivo delays peak bone mass and increases adipogenesis. J. Cell Sci..

[33] Jaiswal R.K., Jaiswal N., Bruder S.P., Mbalaviele G., Marshak D.R., Pittenger M.F. (2000). Adult human mesenchymal stem cell differentiation to the osteogenic or adipogenic lineage is regulated by mitogen-activated protein kinase. J. Biol. Chem..

[34] Cortizo A.M., Lettieri M.G., Barrio D.A., Mercer N., Etcheverry S.B., McCarthy A.D. (2003). Advanced glycation end-products (AGEs) induce concerted changes in the osteoblastic expression of their receptor RAGE and in the activation of extracellular signal-regulated kinases (ERK). Mol. Cell. Biochem..

[35] Hsu Y.L., Liang H.L., Hung C.H., Kuo P.L. (2009). Syringetin, a flavonoid derivative in grape and wine, induces human osteoblast differentiation through bone morphogenetic protein-2/extracellularsignal-regulatedkinase1/2 pathway. Mol. Nutr. Food Res..

[36] Wang D., Ma W., Wang F., Dong J., Wang D., Sun B., Wang B. (2015). Stimulation of Wnt/β-Catenin Signaling to Improve Bone Development by Naringin via Interacting with AMPK and Akt. Cell. Physiol. Biochem..

[37] Huang J., Hsu Y.H., Mo C., Abreu E., Kiel D.P., Bonewald L.F., Brotto M., Karasik D. (2014). METTL21C is a potential pleiotropic gene for osteoporosis sarcopenia acting through the modulation of the NF-κB signaling pathway. J. Bone Miner. Res..

[38] Nguyen M.K., Jeon O., Krebs M.D., Schapira D., Alsberg E. (2014). Sustained localized presentation of RNA interfering molecules from in situ forming hydrogels to guide stem cell osteogenic differentiation. Biomaterials.

[39] Chang N., Xiu L., Li L. (2014). Sphingosine 1-phosphate receptors negatively regulate collagen type I/III expression in human bone marrow-derived mesenchymal stem cell. J. Cell. Biochem..

[40] Poundarik A.A., Diab T., Sroga G.E., Ural A., Boskey A.L., Gundberg C.M., Vashishth D. (2012). Dilatational band formation in bone. Proc. Natl. Acad. Sci. USA.

[41] Akinci B., Bayraktar F., Saklamaz A., Demir T., Yener S., Comlekci A., Ozcan M.A., Kebapcilar L., Yuksel F., Yesil S. (2007). Low transforming growth factor-beta1 serum levels in idiopathic male osteoporosis. J. Endocrinol. Investig..

[42] Qi W.N., Scully S.P. (2000). Extracellular collagen regulates expression of transforming growth factor-beta1 gene. J. Orthop. Res..

[43] Bu S.Y., Lerner M., Stoecker B.J., Boldrin E., Brackett D.J., Lucas E.A., Smith B.J. (2008). Dried plum polyphenols inhibit osteoclastogenesis by downregulating NFATc1 and inflammatory mediators. Calcif. Tissue Int..

[44] Dai J., Patel J.D., Mumper R.J. (2007). Characterization of blackberry extract and its antiproliferative and anti-inflammatory properties. J. Med. Food..

[45] Akagawa M., Shigemitsu T., Suyama K. (2003). Production of hydrogen peroxide by polyphenols polyphenol-rich beverages under quasi-physiological conditions. Biosci. Biotechnol. Biochem..

[46] Gattuso G., Barreca D., Gargiulli C., Leuzzi U., Caristi C. (2007). Flavonoid Composition of Citrus Juices. Molecules.

[47] Fang T., Wang Y., Ma Y., Su W., Bai Y., Zhao P. (2006). A rapid LC/MS/MS quantitation assay for naringin its two metabolites in rat’s plasma. J. Pharm. Biomed. Anal..

[48] Cai Y., Mohseny A.B., Karperien M., Hogendoorn P.C., Zhou G., Cleton-Jansen A.M. (2010). Inactive Wnt/beta-catenin pathway in conventional high-grade osteosarcoma. J. Pathol..

