# Supplementary material for: Bergamot Polyphenol Fraction Exerts Effects on Bone Biology by Activating ERK 1/2 and Wnt/β-Catenin Pathway and Regulating Bone Biomarkers in Bone Cell Cultures
Source: Nutrients. 2018 Sep 14;10(9):1305. doi: 10.3390/nu10091305 (PMC6163325; doi:10.3390/nu10091305)
Supplement: Supplementary file 1 [file nutrients-10-01305-s001.pdf]

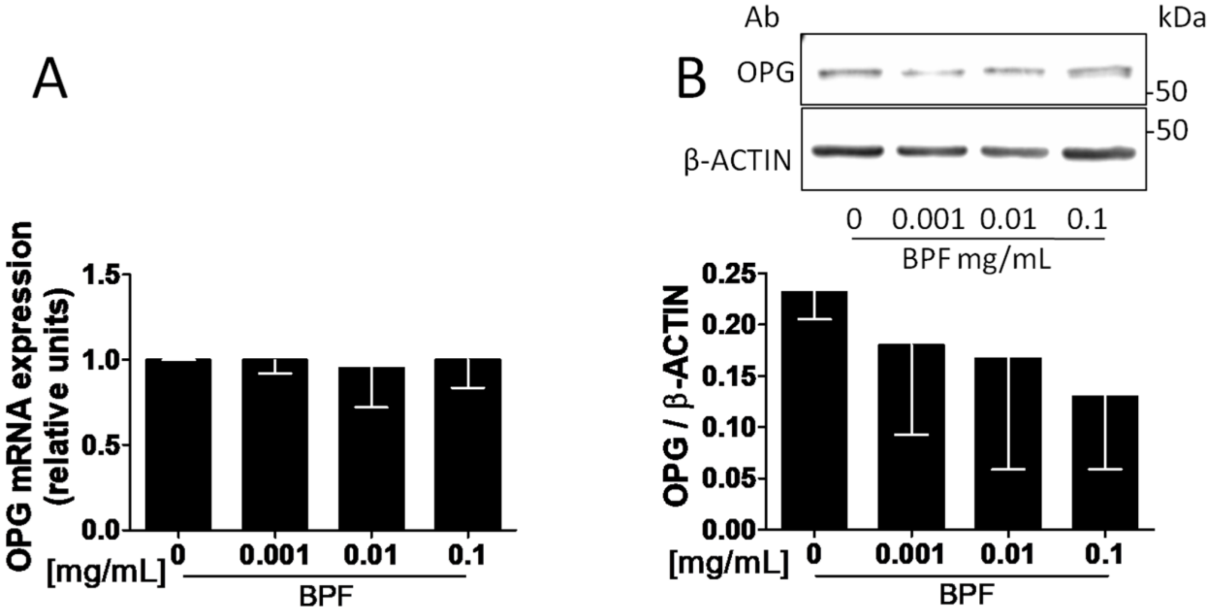

Figure S1. BPF does not increase Osteoprotegerin mRNA and protein levels on Saos-2 cells. Semi-confluent cultures of human osteoblast-like cells (Saos-2) were incubated with BPF 0.001-0.01-0.1 mg/mL for 24 h. (A) mRNA expression levels of Osteoprotegerin were measured by RT-Pcr. Data were analyzed using the $2^{-\Delta\Delta Cq}$ method and normalized to β-actin. (B) Cell proteins were analyzed by Western blotting antibodies specific to Osteoprotegerin and β-actin. Data are represented as mean ± SD. Abbreviations: BPF, Bergamot Polyphenol Fraction.

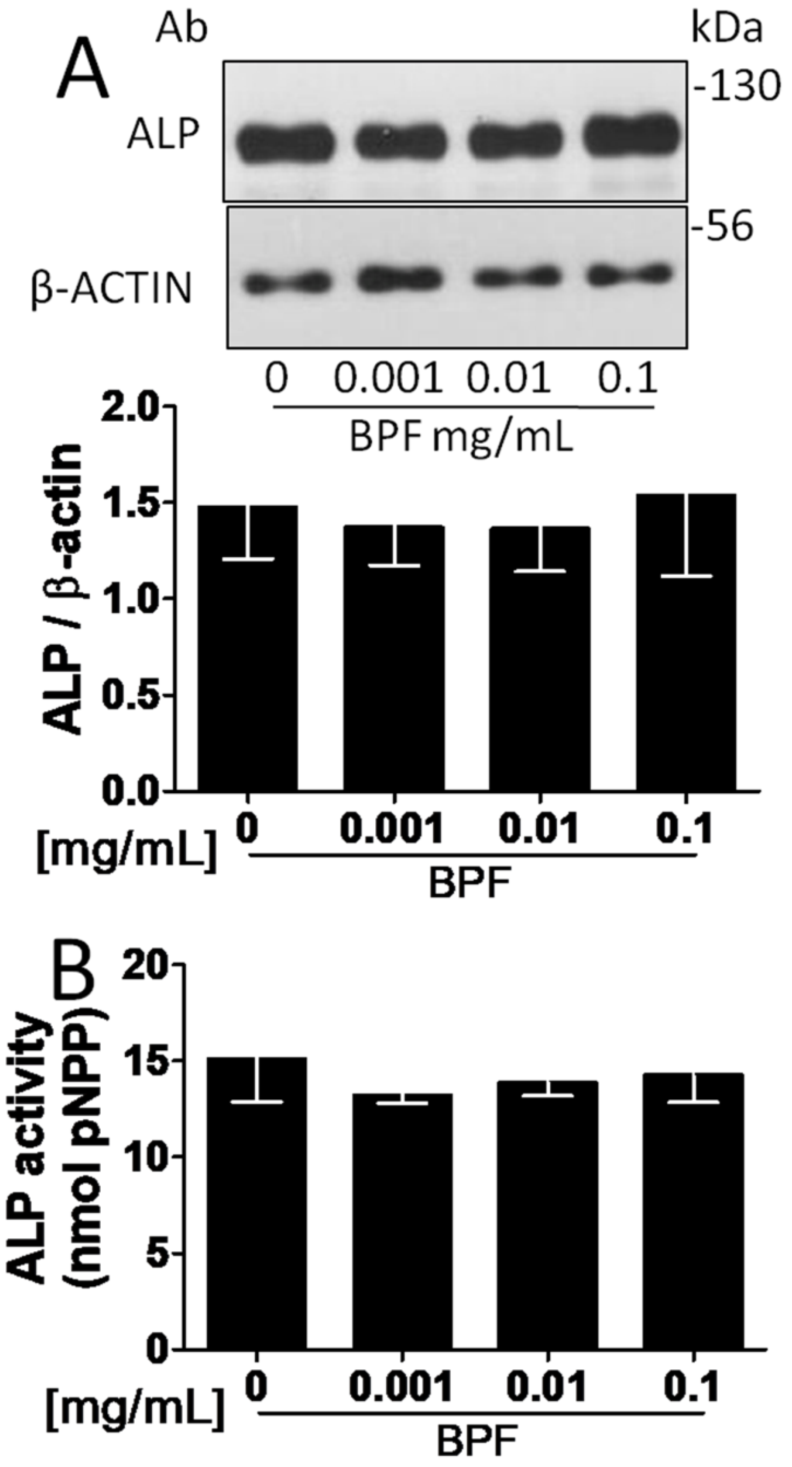

Figure S2. BPF does not increase ALP protein levels and activity on Saos-2 cells. Semi-confluent cultures of human osteoblast-like cells (Saos-2) were incubated with BPF 0.001-0.01-0.1 mg/mL for 24 h. (A) Cell proteins were analyzed by Western blotting antibodies specific to ALP and β-actin. (B) ALP activity was measured by pNPP method. Data are represented as mean ± SD. Abbreviations: BPF, Bergamot Polyphenol Fraction; ALP, Alkaline Phosphatase; pNPP, Para-Nitrophenyl Phosphate.

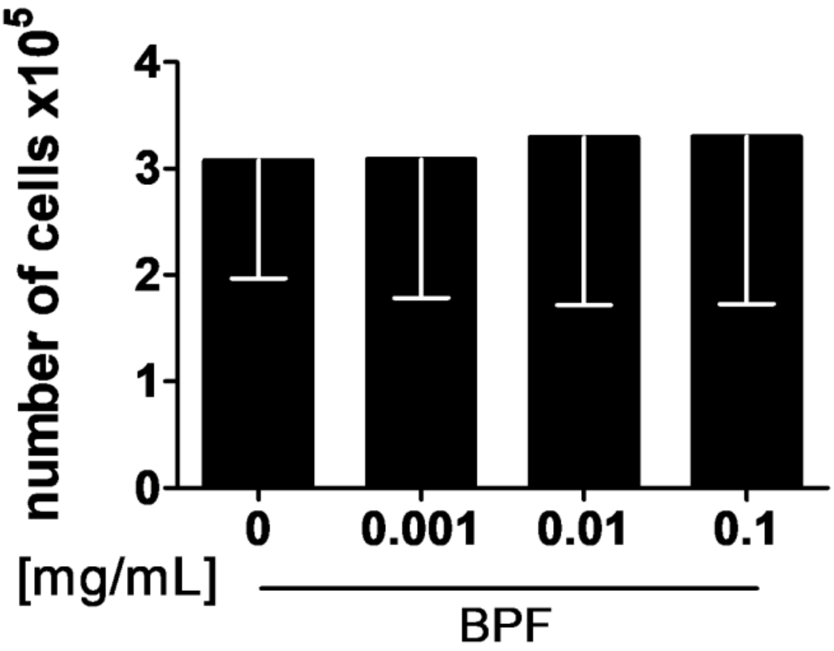

Figure S3. BPF does not increase cell proliferation in MG-63 cells. Semi-confluent cultures of human osteoblast-like cells (MG-63) were incubated with BPF 0.001-0.01-0.1 mg/mL for 24 h. Cell proliferation was determined by counting the number of cells in each well. Data are represented as mean ± SD. Abbreviations: BPF, Bergamot Polyphenol Fraction.
